# Cross-reactivity of steroid hormone immunoassays: clinical significance and two-dimensional molecular similarity prediction

**DOI:** 10.1186/1472-6890-14-33

**Published:** 2014-07-14

**Authors:** Matthew D Krasowski, Denny Drees, Cory S Morris, Jon Maakestad, John L Blau, Sean Ekins

**Affiliations:** 1 Department of Pathology, University of Iowa Hospitals and Clinics, 200 Hawkins Drive, C-671 GH, Iowa, IA 52242, USA; 2 Department of Pathology, University of Michigan, Ann Arbor, MI 48109, USA; 3 Collaborations in Chemistry, Fuquay-Varina, NC 27526, USA

**Keywords:** Anabolic agents, Estradiol, Glucocorticoids, Immunoassays, Progesterone, Similarity, Testosterone

## Abstract

**Background:**

Immunoassays are widely used in clinical laboratories for measurement of plasma/serum concentrations of steroid hormones such as cortisol and testosterone. Immunoassays can be performed on a variety of standard clinical chemistry analyzers, thus allowing even small clinical laboratories to do analysis on-site. One limitation of steroid hormone immunoassays is interference caused by compounds with structural similarity to the target steroid of the assay. Interfering molecules include structurally related endogenous compounds and their metabolites as well as drugs such as anabolic steroids and synthetic glucocorticoids.

**Methods:**

Cross-reactivity of a structurally diverse set of compounds were determined for the Roche Diagnostics Elecsys assays for cortisol, dehydroepiandrosterone (DHEA) sulfate, estradiol, progesterone, and testosterone. These data were compared and contrasted to package insert data and published cross-reactivity studies for other marketed steroid hormone immunoassays. Cross-reactivity was computationally predicted using the technique of two-dimensional molecular similarity.

**Results:**

The Roche Elecsys Cortisol and Testosterone II assays showed a wider range of cross-reactivity than the DHEA sulfate, Estradiol II, and Progesterone II assays. 6-Methylprednisolone and prednisolone showed high cross-reactivity for the cortisol assay, with high likelihood of clinically significant effect for patients administered these drugs. In addition, 21-deoxycortisol likely produces clinically relevant cross-reactivity for cortisol in patients with 21-hydroxylase deficiency, while 11-deoxycortisol may produce clinically relevant cross-reactivity in 11β-hydroxylase deficiency or following metyrapone challenge. Several anabolic steroids may produce clinically significant false positives on the testosterone assay, although interpretation is limited by sparse pharmacokinetic data for some of these drugs. Norethindrone therapy may impact immunoassay measurement of testosterone in women. Using two-dimensional similarity calculations, all compounds with high cross-reactivity also showed a high degree of similarity to the target molecule of the immunoassay.

**Conclusions:**

Compounds producing cross-reactivity in steroid hormone immunoassays generally have a high degree of structural similarity to the target hormone. Clinically significant interactions can occur with structurally similar drugs (e.g., prednisolone and cortisol immunoassays; methyltestosterone and testosterone immunoassays) or with endogenous compounds such as 21-deoxycortisol that can accumulate to very high concentrations in certain disease conditions. Simple similarity calculations can help triage compounds for future testing of assay cross-reactivity.

## Background

Immunoassays are frequently used in laboratory medicine for quantitation of plasma/serum concentrations of steroid hormones such as cortisol, dehydroepiandrosterone (DHEA) sulfate, estradiol, progesterone, or testosterone [1,2]. A variety of immunoassay methods are available, ranging from enzyme-linked immunosorbent assays (ELISAs) to homogeneous immunoassays that can be run on high-throughput analyzers commonly found in hospital clinical laboratories [3]. The most common alternative approach for measurement of steroid hormones is chromatography (especially high-performance liquid chromatography, HPLC), either alone or in combination with mass spectrometry (MS) (HPLC/MS or simply LC/MS) [4-7]. An increasing number of clinical laboratories, particularly reference laboratories, utilize liquid chromatography-tandem mass spectrometry (LC/MS/MS) for steroid hormone measurement [5]. LC/MS/MS provides a high degree of specificity for steroid hormone measurement. However, chromatography and mass spectrometry require specialized technical skill along with dedicated and often expensive instrumentation. Consequently, many clinical laboratories continue to use immunoassays for routine steroid hormone measurement.

One limitation of steroid hormone immunoassays is interference caused by compounds with structural similarity to the target steroid molecule against which the assay antibodies were generated [8]. Interfering molecules can be structurally related endogenous compounds (e.g., 6β-hydroxycortisol for a cortisol assay), drugs (including anabolic steroids and herbal medications), or natural products. Metabolites of these compounds may additionally cross-react. The manufacturers of commercially marketed steroid hormone immunoassays test a variety of endogenous and synthetic hormones for cross-reactivity and report this data in the assay package insert, typically as percent cross-reactivity or sometimes qualitatively (e.g., using a descriptor such as “cross-reacts”). The extent of cross-reactivity testing varies by assay and manufacturer without a clearly defined standard for which and how many compounds to test [8,9].

While the package inserts or other manufacturers’ documents collectively contain extensive data on marketed steroid hormone immunoassay cross-reactivity, studies of previously unreported interference with steroid hormone immunoassays have also appeared in the scientific literature. Examples include cross-reactivity of DHEA sulfate with testosterone immunoassays [10,11], mifepristone with estradiol and testosterone enzyme immunoassays [12], and methylprednisolone with cortisol immunoassays [13].

In this study, we determine cross-reactivity of a variety of steroid and steroidal compounds (including anabolic steroids) to the Roche Diagnostics Elecsys immunoassays for cortisol, DHEA sulfate, estradiol, progesterone, and testosterone, and compare these results to other cross-reactivity studies. We additionally utilize computational methodology to attempt to predict cross-reactivity of compounds for steroid hormone immunoassays, building on previous work using similarity analysis to predict cross-reactivity of drug of abuse and therapeutic drug monitoring assays [14-17]. Our hypothesis is that a given compound is more likely to cross-react with an immunoassay if the compound shares a high level of structural similarity to the target molecule/hapten of the assay. To our knowledge, similarity analysis has not been applied to the prediction of cross-reactivity of immunoassays used to measure steroid hormones. Lastly, we discuss cross-reactivity mostly likely to have impact on clinical testing using steroid hormone immunoassays.

## Methods

### Chemicals

Test compounds were obtained from Steraloids (Providence, Rhode Island, USA) or Sigma-Aldrich (St. Louis, Missouri, USA) (see Additional file 1 for sources and product numbers for all compounds tested). Interference studies were designed from NCCLS Guideline EP7-A [18], with test compounds spiked into normal human plasma. Each spiked sample was compared to the unadulterated sample, with the degree of interference expressed as percent cross-reactivity.

### Cross-reactivity Studies

The assays tested experimentally were the Roche Diagnostics (Indianapolis, IN, USA) Cortisol, DHEA sulfate, Estradiol II, Progesterone II, and Testosterone II assays for Elecsys and Modular E170 analyzers. All assays were run using manufacturer specifications on Modular E170 analyzers. The complete details on assays are in the Additional file 1. Cross-reactivity was grouped into four broad categories: Strong Cross-Reactivity (5% or greater), Weak Cross-Reactivity (0.5-4.9%), Very Weak Cross-Reactivity (0.05-0.49%), and Not Cross-Reactive (<0.05%). Percent cross-reactivity is defined as the ratio of observed “steroid” to the amount of test compound added, multiplied by 100. These categories do not imply clinical significance but provide a broad framework to compare degree of cross-reactivity.

To estimate clinical significance of cross-reactivity, a literature search was conducted for studies that have reported plasma/serum concentrations of the potentially cross-reactive compounds. These included pharmacokinetic studies of steroidal drugs or investigations of steroid hormone concentrations in disorders such as 11β-hydroxylase or 21-hydroxylase deficiency. These references are reported in Tables 1, 2, 3, 4 and 5.

**Table 1:** Cortisol immunoassay cross-reactivity

**Compound**	**Plasma/serum concentrations**	**Cross-reactivity in Roche assay**	**Likelihood of clinically significant cross-reactivity**
Cortisol (endogenous compound)	• 62 – 194 ng/mL (morning) [19]	100%	High (assay target)
	• 23 – 119 ng/mL (afternoon) [19]		
6-Methylprednisolone	Up to 1,000 ng/mL after oral or intravenous administration [20]	249%	High
Allotetrahydrocortisol	Unknown	165% (package insert)	Unknown, serum/plasma concentrations not reported
6β-Hydroxycortisol	Unknown	158% (package insert)	Unknown, serum/plasma concentrations not reported
Prednisolone	Up to 400 ng/mL in pediatric transplant patients [21]	148%	High
21-Deoxycortisol	• 0.28 – 0.43 ng/mL (pediatric controls) [22]	45.4% (package insert)	Low, except in patients with 21-hydroxylase deficiency
	• Up to 140 ng/mL (patients with 21-hydroxylase deficiency) [22]		
Fludrocortisone	• 0.36 ng/ml after single dose [23]	7.7%	Low
5β-Dihydrocorticosterone	Unknown	4.9%	Unknown, serum/plasma concentrations not reported
Corticosterone	• 0.18 – 2.0 ng/mL (18 years and younger) [24]	4.6%	Low
	• 0.53 – 1.6 ng/mL (<18 years) [24]		
11-Deoxycortisol	• 0.17 – 1.8 ng/mL (pediatric controls) [22]	4.6%	Low, except in patients following metyrapone challenge or who have 11β-hydroxylase deficiency
	• Up to 63 ng/mL (patients with 11β-hydroxylase deficiency) [22]		
	• Up to 250 ng/mL (following metyrapone challenge) [25]		
Canrenone	10 – 1,000 ng/mL in patients receiving spironolactone [26-28]	1.8%	Low, except if cortisol measured during peak canrenone concentrations
17-Hydroxyprogesterone	• 0.08 – 2.0 ng/mL (pediatric controls) [22]	1.6%	Low, except in patients with 21-hydroxylase deficiency
	• Up to 1,005 ng/mL (21-hydroxylase deficiency) [22]		
Formestane	Up to 14 ng/mL in breast cancer patients [29]	1.2%	Low
Androstenedione	• Up to 0.86 ng/mL before onset of puberty [30]	0.9%	Low
	• Up to 3.2 ng/mL in 21-hydroxylase deficiency [30]		
Prednisone	Up to 57 ng/mL in pediatric transplant patients receiving prednisolone [21]	0.3%	Low

**Table 2:** DHEA sulfate immunoassay cross-reactivity

**Compound**	**Plasma/serum concentrations**	**Cross-reactivity in Roche assay**	**Likelihood of clinically significant cross-reactivity**
DHEA sulfate	• Up to 6,070 ng/mL (infants) [31]	100%	High (assay target)
	• 5 – 4,070 ng/mL (females > 1 year old) [31]		
	• 5–4,920 ng/mL (males > 1 year old) [31]		
Pregnenolone sulfate	• 21 – 84 ng/mL (adult controls) [32]	2.1%	Low, possible minor effect around time of parturition.
	• Up to 1,580 ng/mL in pregnancy [33]		
17-Hydroxyprogesterone	• 0.1 – 2.0 ng/mL (pediatric controls) [22]	0.08%	Low, possible minor effect in 21-hydroxylase deficiency.
	• Up to 1,005 ng/mL (21-hydroxylase deficiency) [22]		
17-Hydroxypregnenolone	• Up to 4.3 ng/ml in healthy females [34,35]	0.05%	Low
	• Up to 4.8 ng/mL in healthy males [34,35]		
	• Up to 100 ng/mL in premature infants [36]		

**Table 3:** Estradiol immunoassay cross-reactivity

**Compound**	**Plasma/serum concentrations**	**Cross-reactivity in Roche assay**	**Likelihood of clinically significant cross-reactivity**
Estradiol (endogenous compound)	• 0.01 – 0.04 ng/mL (males) [37]	100%	High (assay target)
	• 0.013 – 0.50 ng/mL (premenopausal females) [37]		
	• Up to 4.3 ng/mL (pregnancy) [37]		
Estrone	• Up to 0.06 ng/mL (males) [24]	0.54%	Low
	• Up to 0.2 ng/mL (females) [24]		
Ethinyl estradiol	Up to 0.1 ng/mL while on medication [38-41]	0.23%	Low
Estriol	• Up to 18 ng/mL in pregnancy [42]	0.09%	Possible contribution in pregnancy
	• Up to 2.4 ng/mL in non-pregnant females [43]		

**Table 4:** Progesterone immunoassay cross-reactivity

**Compound**	**Plasma/serum concentrations**	**Cross-reactivity in Roche assay**	**Likelihood of clinically significant cross-reactivity**
Progesterone	• 0.2 – 1.4 ng/mL (males) [44]	100%	High (assay target)
	• Up to 27 ng/mL (women) [44]		
5β-Dihydroprogesterone	Up to 0.8 ng/mL in adults [45]	18.2%	Possible significant contribution for individuals with progesterone concentrations on the lower end of reference interval
17-Hydroxyprogesterone	• 0.08 – 2.0 ng/mL (pediatric controls) [22]	1.2%	Low, except in patients with 21-hydroxylase deficiency
	• Up to 1,005 ng/mL (21-hydroxylase deficiency) [22]		
Pregnanolone	Up to 17 ng/mL in women [33]	0.90%	Low
Allopregnanolone	Up to 29 ng/mL in women [33]	0.82%	Low
Medroxyprogesterone	Up to 100 ng/mL following dosing in women [46]	0.67%	Possible significant contribution for individuals with progesterone concentrations on the lower end of reference interval
Corticosterone	• 0.18 – 2.0 ng/mL (18 years and younger) [24]	0.54%	Low
	• 0.53 – 1.6 ng/mL (<18 years) [24]		
11-Deoxycortisol	• 0.17 – 1.8 ng/mL (pediatric controls) [22]	0.39%	Low, except in patients following metyrapone challenge or who have 11β-hydroxylase deficiency
	• Up to 63 ng/mL (11β-hydroxylase deficiency) [22]		
	• Up to 250 ng/mL (metyrapone challenge) [25]		
Nandrolone	Up to 5.16 ng/mL in men following intramuscular injection [47]	0.17%	Low
Pregnenolone	Up to 3.27 ng/mL in women [33]	0.12%	Low
Exemestane	Up to 441 ng/mL in post-menopausal women [48]	0.09%	Possible significant effect if progesterone measured near peak of exemestane plasma concentration
Androstenedione	• Up to 0.86 ng/mL before onset of puberty [30]	0.09%	Low
	• Up to 3.2 ng/mL in 21-hydroxylase deficiency [30]		

**Table 5:** Testosterone immunoassay cross-reactivity

**Compound**	**Plasma/serum concentrations**	**Cross-reactivity in Roche assay**	**Likelihood of clinically significant cross-reactivity**
Testosterone (endogenous compound)	• 0 – 10 ng/mL (males) [49]	100%	High (assay target)
	• 0 – 0.5 ng/mL (females) [49]		
Methyltestosterone	Up to 40 ng/mL following single dose [50]	12.2%	High, especially if measured during peak concentration
Boldenone	Up to 1.1 ng/mL in horses following dosing [51]	7.2%	Likely low, although there is lack of human pharmacokinetic data
19-Norclostebol	Unknown	6.7%	Unknown, no human pharmacokinetic data available
Norethindrone	Up to 20 ng/mL while on medication [39-41]	6.7%	Possible significant contribution in women taking norethindrone, especially if specimen drawn near peak of norethindrone concentration
11β-Hydroxytestosterone	Unknown	5.5%	Unknown, no human pharmacokinetic data available
Methandrostenolone	Unknown	5.4%	Unknown, no human pharmacokinetic data available
Normethandrolone	Unknown	5.4%	Unknown, no human pharmacokinetic data available
Nandrolone	Up to 5.16 ng/mL [47]	2.1%	Low
Androstenedione	• Up to 0.86 ng/mL before onset of puberty [30]	1.2%	Low
	• Up to 3.2 ng/mL in 21-hydroxylase deficiency [30]		

### Two-dimensional (2D) Similarity analysis

**Figure 1:**
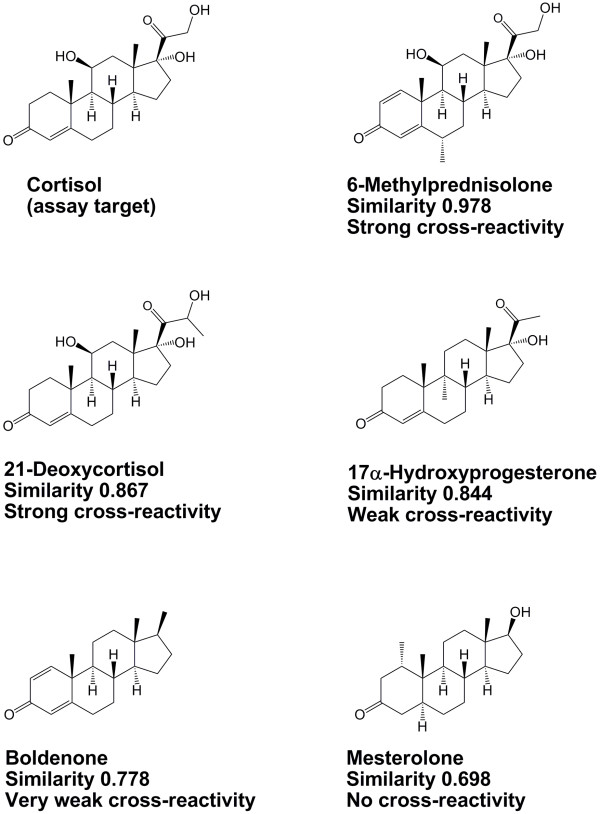
**Illustration of two-dimensional similarity to cortisol.** The figure depicts chemical structures of cortisol, 6-methylprednisolone, 21-deoxycortisol, 17α-hydroxyprogesterone, boldenone, and mesterolone. Below each compound is the two-dimensional similarity to cortisol and the degree of cross-reactivity to the Roche Elecsys Cortisol immunoassay.

Comparison of similarity of test molecules to the target compounds of the steroid immunoassays used two-dimensional (2D) similarity analysis, which determines the similarity between molecules independent of any *in vitro* data [52-54]. These methods have been applied in our previous publications on cross-reactivity of drug of abuse and therapeutic drug monitoring immunoassays [14-17]. 2D similarity searching used the “find similar molecules by fingerprints” protocol in Discovery Studio versions 2.5.5 and 3.5 (Accelrys, Inc., San Diego, California, USA). MDL public keys (a specific 2D similarity algorithm) were used with the Tanimoto similarity coefficient (ranging from 0 to 1 with 1 being maximally similar and 0 being maximally dissimilar) and an input query. It should be noted that 2D similarity algorithms do not distinguish between diastereomers and enantiomeric pairs. 2D similarity for each test compound was compared to the target molecule of the immunoassay (e.g., estradiol or progesterone) undergoing analysis. Figure 1 illustrates 2D similarity of five compounds to cortisol.

## Results

### Cortisol immunoassay

Based on package insert data and the experimental testing in the current study, six compounds produce cross-reactivity of 5% of greater on the Roche Elecsys Cortisol assay at a test concentration of 1 μg/mL (1,000 ng/mL): 6β-hydroxycortisol, allotetrahydrocortisol, 21-deoxycortisol, fludrocortisone, prednisolone, and 6-methylprednisolone (Figure 1; Table 1; Additional file 1). Seventeen additional compounds produced cross-reactivity between 0.5 and 4.9%.

**Figure 2:**
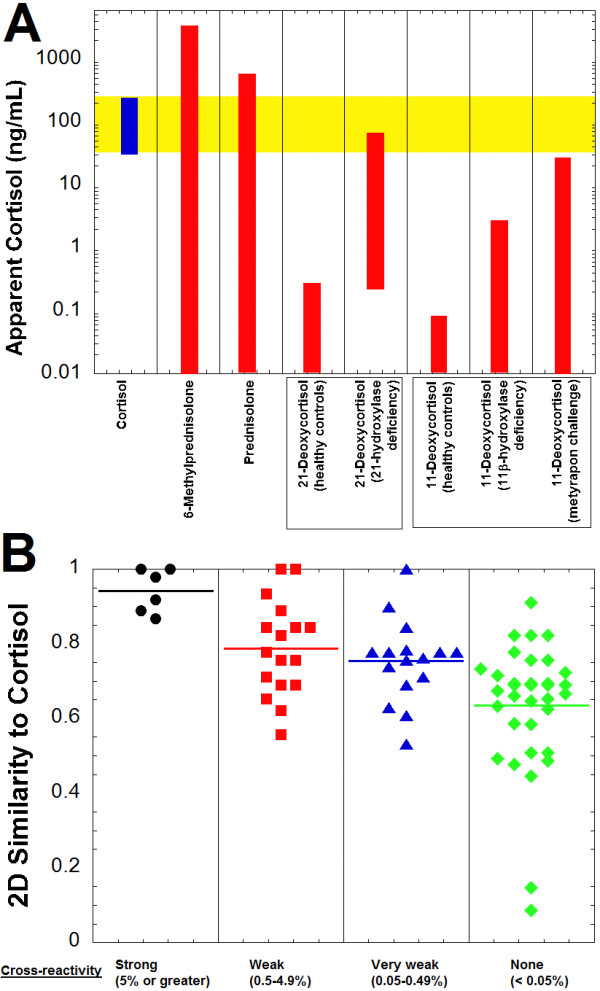
**Cortisol immunoassay cross-reactivity and similarity predictions. A**. The plot shows the cortisol reference range for adults (highlighted in yellow) in comparison to the predicted apparent cortisol concentrations produced on the Roche Elecsys Cortisol assay by 6-methylprednisolone, prednisolone, 21-deoxycortisol (healthy controls and patients with 21-hydroxylase deficiency), and 11-deoxycortisol (healthy controls, patients with 11β-hydroxylase deficiency, and following metyapon challenge). Table 1 contains the concentration ranges and percent cross-reactivity values from which the estimated apparent cortisol concentrations are derived. **B**. Two-dimensional similarity of compounds to cortisol is shown, sorted by degree of cross-reactivity in the Roche Cortisol assay (horizontal line in each column indicates average similarity within that group). Similarity values vary from 0 to 1, with 1 being maximally similar. The compounds are subdivided into categories of strong cross-reactivity (5% or greater, black circles), weak cross-reactivity (0.5-4.9%, red squares), very weak cross-reactivity (0.05-0.49%, blue triangles), and no cross-reactivity (<0.05%, green diamonds) to the Roche Cortisol assay (complete list of compounds and associated cross-reactivities and 2D similarities is in Additional file 1).

Using the cross-reactivity values, the apparent cortisol concentration that could be produced on the Roche Elecsys immunoassay was estimated for compounds based on published serum/plasma concentrations, if available (Figure 2A, Table 1). Prednisolone and 6-methylprednisolone are both predicted to produce substantial apparent cortisol concentrations on the Roche immunoassay at serum/plasma concentrations typical in patients administered these drugs. Falsely elevated cortisol readings may also occur with 21-deoxycortisol in patients with 21-hydroxylase deficiency and with 11-deoxycortisol following metyrapone challenge (Figure 2A, Table 1). In both scenarios, clinically significant falsely elevated cortisol measurements are most likely only when 21-deoxycortisol or 11-deoxycortisol are at the high end of serum/plasma concentrations reported in the literature.The majority of the compounds with strong cross-reactivity for the Roche Elecsys Cortisol assay had 2D-similarities to cortisol of 0.867 or higher (Figure 2B). Only one compound (tetrahydrocortisone) had a 2D-similarity to cortisol higher than 0.867 but was not cross-reactive in our study.

### DHEA sulfate immunoassay

No compounds produced greater than 5% cross-reactivity on the Roche Elecsys DHEA sulfate immunoassay at a test concentration of 50 μg/mL (50,000 ng/mL), and only two compounds (estropipate and pregnenolone sulfate) produced greater than 0.5% cross-reactivity (Table 2; Additional file 1). Twenty-six compounds produced cross-reactivity between 0.05 and 0.5%. This group of compounds with very weak cross-reactivity included anabolic steroids (nandrolone), androstanes (androstenedione, androsterone sulfate), estranes (estrone-3-sulfate), and pregnanes (17α-hydroxypregnenolone).

**Figure 3:**
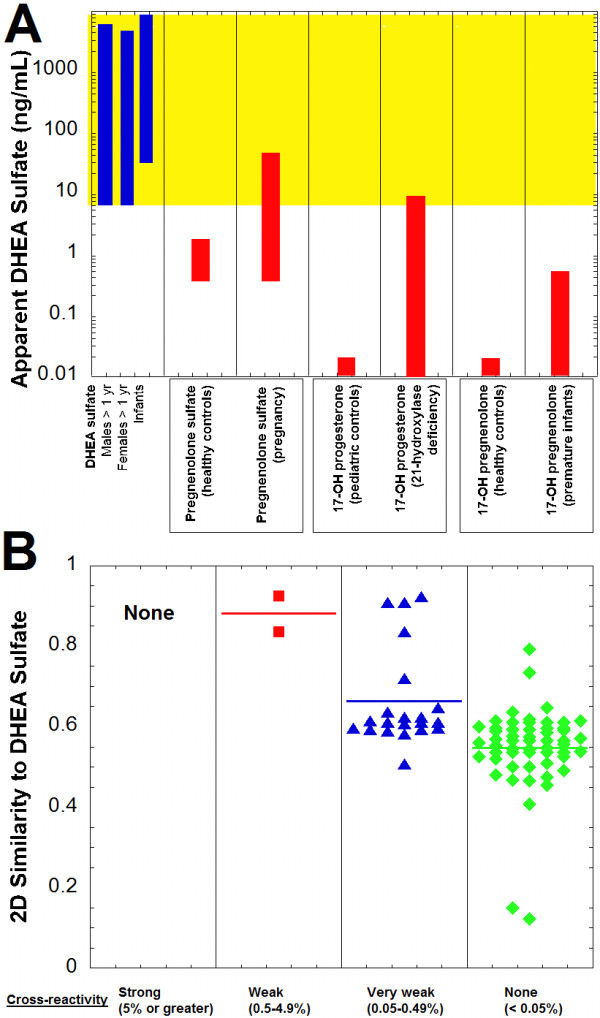
**DHEA sulfate immunoassay cross-reactivity and similarity predictions. A**. The plot shows the DHEA sulfate reference range for males greater than 1 year old, females greater than 1 year old, and infants in comparison to the predicted apparent DHEA sulfate concentrations produced on the Roche Elecsys DHEA sulfate assay by pregnenolone sulfate (healthy controls and in pregnancy), 17-hydroxyprogesterone (pediatric controls and patients with 21-hydroxylase deficiency), and 17-hydroxypregnenolone (healthy controls and premature infants). Table 2 contains the concentration ranges and percent cross-reactivity values from which the estimated apparent DHEA sulfate concentrations are derived. **B**. Two-dimensional similarity of compounds to DHEA sulfate is shown, sorted by degree of cross-reactivity in the Roche DHEA sulfate assay (horizontal line in each column indicates average similarity within that group). Similarity values vary from 0 to 1, with 1 being maximally similar. The compounds are subdivided into categories of strong cross-reactivity (5% or greater, black circles), weak cross-reactivity (0.5-4.9%, red squares), very weak cross-reactivity (0.05-0.49%, blue triangles), and no cross-reactivity (<0.05%, green diamonds) to the Roche DHEA sulfate assay (complete list of compounds and associated cross-reactivities and 2D similarities is in Additional file 1).

Using the cross-reactivity values, the apparent DHEA sulfate concentration that could be produced on the Roche Elecsys immunoassay was estimated for compounds based on published serum/plasma concentrations, if available (Figure 3A, Table 2). In only two scenarios was the apparent DHEA sulfate concentration predicted to fall within the reference ranges for DHEA sulfate – pregnenolone sulfate in pregnancy and 17-hydroxyprogesterone in patients with 21-hydroxylase deficiency (Figure 3A).

The two compounds with the highest percent cross-reactivity in the DHEA sulfate immunoassay (estropipate and pregnenolone sulfate) had higher 2D similarities to DHEA sulfate than all 56 compounds classified as non-cross-reactive (Figure 3B; Additional file 1). Only 4 of 20 compounds with very weak cross-reactivity has 2D similarity to DHEA sulfate greater than 0.8. All non-cross-reactive compounds had 2D similarities less than 0.8.

### Estradiol immunoassay

Only estrone (0.54%) produced greater than 0.5% cross-reactivity on the Roche Elecsys Estradiol II immunoassay at a challenge of 1 μg/mL (1,000 ng/mL) (Table 3; Additional file 1). Estriol, estropipate, ethinyl estradiol, 2-methoxy-estradiol, 17β-estradiol-17-valerate, and 17β-estradiol-3, 17-disulfate each produced very weak cross-reactivity between 0.05 and 0.5%. The aromatase inhibitors exemestane, formestane, and letrozole produced no detectable cross-reactivity.

**Figure 4:**
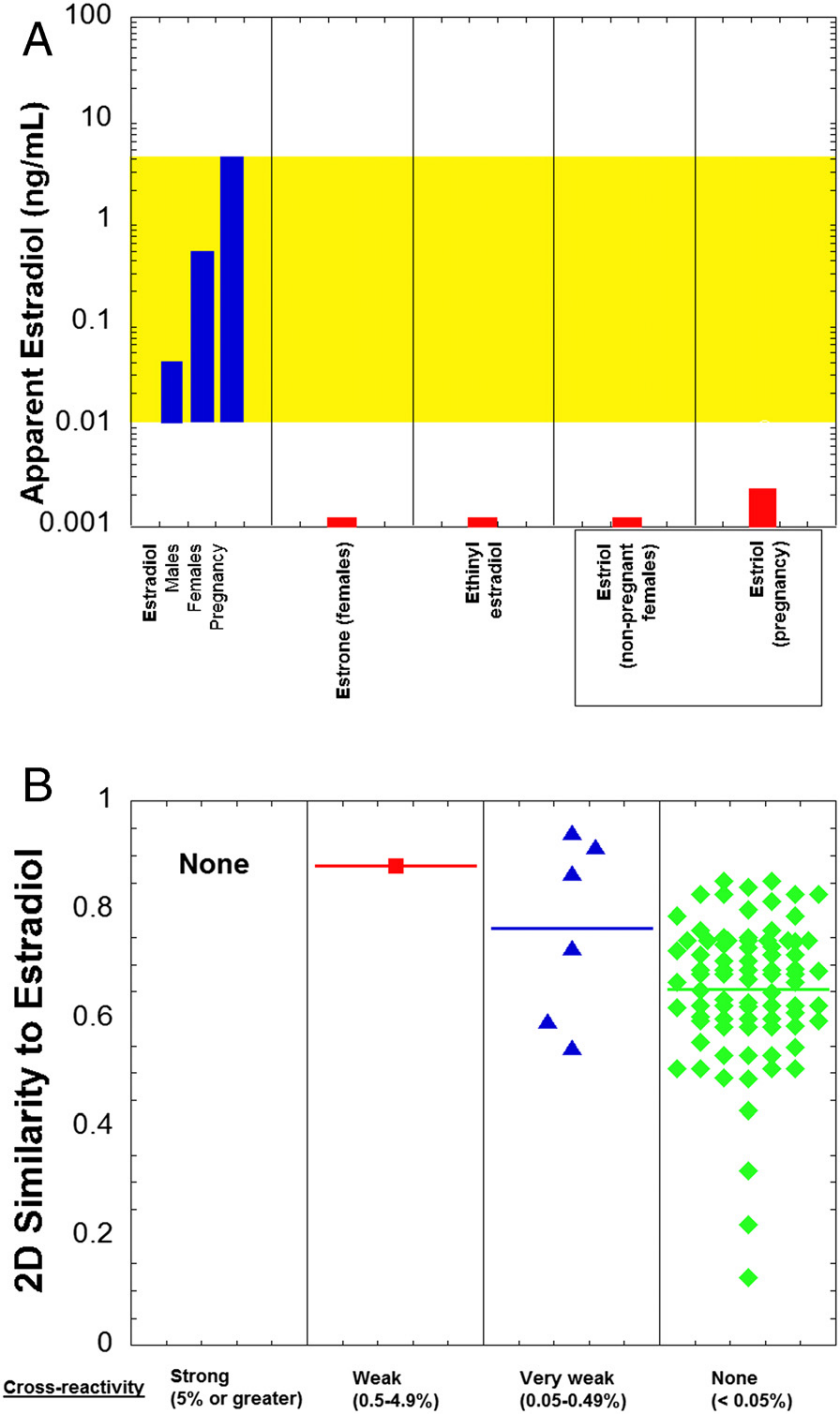
**Estradiol immunoassay cross-reactivity and similarity predictions. A**. The plot shows the estradiol reference ranges for males, non-pregnant females, and pregnant females (highlighted in yellow) in comparison to the predicted apparent estradiol concentrations produced on the Roche Elecsys Estradiol II assay by estrone (females), ethinyl estradiol, and estriol (non-pregnant and pregnant females). Table 3 contains the concentration ranges and percent cross-reactivity values from which the estimated apparent estradiol concentrations are derived. **B**. Two-dimensional similarity of compounds to estradiol is shown, sorted by degree of cross-reactivity in the Roche Estradiol II assay (horizontal line in each column indicates average similarity within that group). Similarity values vary from 0 to 1, with 1 being maximally similar. The compounds are subdivided into categories of strong cross-reactivity (5% or greater, black circles), weak cross-reactivity (0.5-4.9%, red squares), very weak cross-reactivity (0.05-0.49%, blue triangles), and no cross-reactivity (<0.05%, green diamonds) to the Roche Estradiol II assay (complete list of compounds and associated cross-reactivities and 2D similarities is in Additional file 1).

Using the cross-reactivity values, the apparent estradiol concentration that could be produced on the Roche Elecsys immunoassay was estimated for compounds based on published serum/plasma concentrations, if available (Figure 4A, Table 3). No compound was predicted to produce estradiol concentrations within the reference range for males or females. Even estriol, which can reach high concentrations in pregnancy, likely produces little or no clinically significant impact on the Roche estradiol immunoassay due to low cross-reactivity. The 2D similarities of estrone (0.882), ethinyl estradiol (0.943), and estriol (0.917) were higher than any of the compounds that were not cross-reactive on the Roche assay (Figure 4B).

### Progesterone immunoassay

5β-Dihydroprogesterone (5β-pregnan-3,20-dione) was the most cross-reactive compound for the Roche Elecsys Progesterone II immunoassay at a challenge of 1 μg/mL (1,000 ng/mL), with a cross-reactivity of 18.2% (Table 4; Additional file 1). 17-Hydroxyprogesterone, 5α-pregnan-3-ol-20-one, 5α-pregnan-3,20-dione, 5α-pregnenolone, medroxyprogesterone, and pregnanolone each produced weak cross-reactivity between 0.5% and 4.9%. An additional twenty-two compounds produced very weak cross-reactivity (0.05-0.49%).

**Figure 5:**
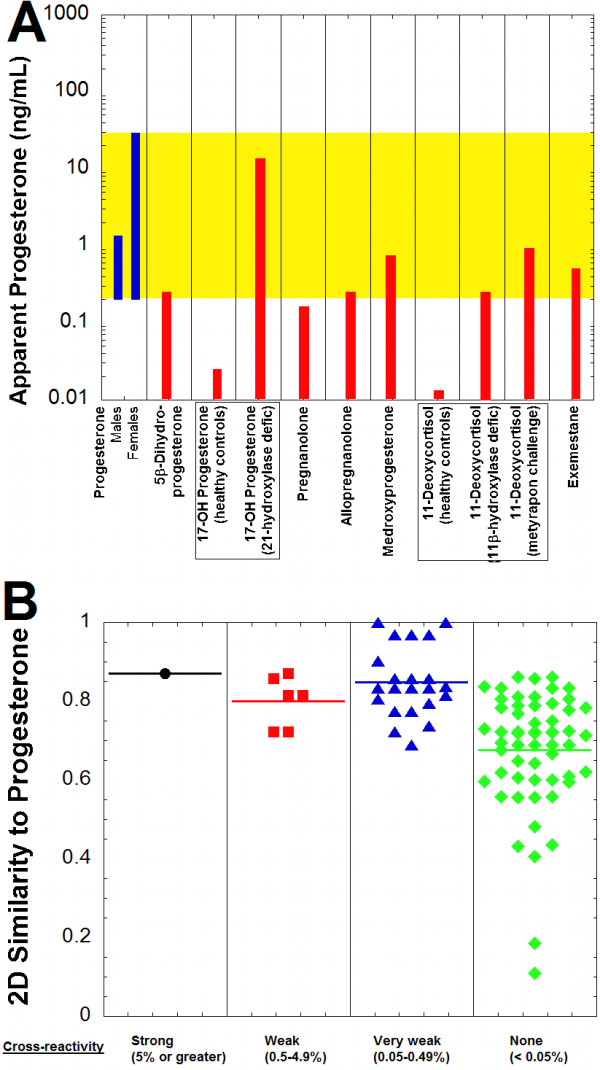
**Progesterone immunoassay cross-reactivity and similarity predictions. A**. The plot shows the progesterone reference range for adult males and females (highlighted in yellow) in comparison to the predicted apparent progesterone concentrations produced on the Roche Elecsys Progesterone II assay by 5β-dihydroprogesterone, 17-hydroxyprogesterone (pediatric controls and patients with 21-hydroxylase deficiency), pregnanolone, allopregnanolone, medroxyprogesterone, 11-deoxycortisol (healthy controls, patients with 11β-hydroxylase deficiency, and following metyapon challenge), and exemestane. Table 4 contains the concentration ranges and percent cross-reactivity values from which the estimated apparent progesterone concentrations are derived. **B**. Two-dimensional similarity of compounds to progesterone is shown, sorted by degree of cross-reactivity in the Roche Progesterone II assay (horizontal line in each column indicates average similarity within that group). Similarity values vary from 0 to 1, with 1 being maximally similar. The compounds are subdivided into categories of strong cross-reactivity (5% or greater, black circles), weak cross-reactivity (0.5-4.9%, red squares), very weak cross-reactivity (0.05-0.49%, blue triangles), and no cross-reactivity (<0.05%, green diamonds) to the Roche Progesterone II assay (complete list of compounds and associated cross-reactivities and 2D similarities is in Additional file 1).

Using the cross-reactivity values, the apparent progesterone concentration that could be produced on the Roche Elecsys immunoassay was estimated for compounds based on published serum/plasma concentrations, if available (Figure 5A, Table 4). The most significant apparent progesterone concentrations were estimated to occur with 17-hydroxyprogesterone in patients with 21-hydroxylase deficiency and for 11-deoxycortisol following metyrapone challenge. 5β-Dihydroprogesterone and allopregnanolone both may produce apparent progesterone concentrations of approximately 0.2 ng/mL, but likely only when these compounds are the highest end of what can occur physiologically (Table 4). Two medications, medroxyprogesterone and exemestane, each may have cross-reactivity on the progesterone immunoassay that can produce apparent progesterone concentration of approximately 0.5 ng/mL.

5β-Dihydroprogesterone had higher 2D similarity to progesterone than any compound that was not cross-reactive (Figure 5B; Additional file 1). All compounds with strong or weak cross-reactivity had 2D similarities of 0.722 or higher to progesterone. In contrast, only 27 of 55 compounds that were non-cross-reactive on the Roche progesterone immunoassay had 2D similarity of 0.722 or higher to progesterone.

### Testosterone immunoassay

Anabolic steroids were well-represented among compounds cross-reacting with the Roche Elecsys Testosterone II immunoassay at a test concentration of 0.1 μg/mL (100 ng/mL) (Table 5; Additional file 1). Seven compounds (boldenone, 19-norclostebol, dianabol, methyltestosterone, norethindrone, normethandrolone, and 11β-hydroxytestosterone) produced cross-reactivity of 5% or greater. Nine additional compounds produced cross-reactivity between 0.5% and 4.9%. Examples of anabolic steroids that produced no detectable cross-reactivity included anasterone (oxymetholone), stanozolol, and turinabol.

**Figure 6:**
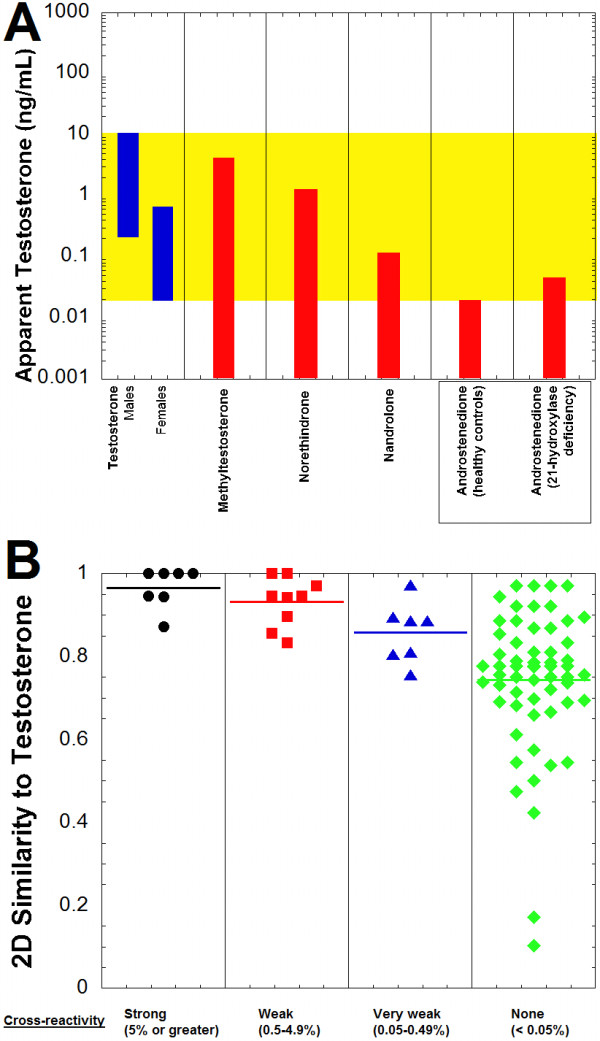
**Testosterone immunoassay cross-reactivity and similarity predictions. A**. The plot shows the testosterone reference range for males and females (highlighted in yellow) in comparison to the predicted apparent testosterone concentrations produced on the Roche Elecsys Testosterone II assay by methyltestosterone, norethindrone, nandrolone, and androstenedione (healthy controls and patients with 21-hydroxylase deficiency). Table 5 contains the concentration ranges and percent cross-reactivity values from which the estimated apparent testosterone concentrations are derived. **B**. Two-dimensional similarity of compounds to testosterone is shown, sorted by degree of cross-reactivity in the Roche Testosterone II assay (horizontal line in each column indicates average similarity within that group). Similarity values vary from 0 to 1, with 1 being maximally similar. The compounds are subdivided into categories of strong cross-reactivity (5% or greater, black circles), weak cross-reactivity (0.5-4.9%, red squares), very weak cross-reactivity (0.05-0.49%, blue triangles), and no cross-reactivity (<0.05%, green diamonds) to the Roche Testosterone II assay (complete list of compounds and associated cross-reactivities and 2D similarities is in Additional file 1).

Using the cross-reactivity values, the apparent testosterone concentration that could be produced on the Roche Elecsys Testosterone II immunoassay was estimated for compounds based on published serum/plasma concentrations, if available (Figure 6A, Table 5). There is limited published data on serum/plasma concentrations of some of the anabolic steroids, with generally more focus on measurement of these compounds in urine samples, usually for the purposes of detecting use as performance-enhancing drugs in competitive athletics [55-57]. Of the anabolic steroids for which serum/plasma concentrations are available, methyltestosterone appears to be the one most likely to impact testosterone immunoassay measurements in males. Norethindrone and nandrolone could produce clinically significant impact on testosterone measurement in women, as may androstenedione in patients with 21-hydroxylase deficiency (Figure 6A, Table 5).All but one compound (testosterone propionate) that had cross-reactivity of 0.05% or greater on the Roche assay had 2D similarities to testosterone of 0.8 or greater (Figure 6B). Only 20 of 59 compounds that were non-cross-reactive on the Roche testosterone immunoassay had 2D similarity of 0.8 or higher to testosterone.

## Discussion

Immunoassays are commonly used clinically for measurement of steroid hormone serum/plasma concentrations. In many situations, immunoassays produce results comparable to the more specific chromatography/mass spectrometry-based methods. However, a growing number of studies have documented differences between immunoassays and mass spectrometry methods (especially LC/MS/MS) [6,7,58-63]. Some differences can be attributed to lower limit of quantitation issues with immunoassays, especially for analytes that may be very low in concentration in some populations (e.g., testosterone in females or estradiol in males). Cross-reactivity to endogenous or exogenous compounds other than the target steroid hormone of the assay may also contribute to differences between immunoassay and LC/MS/MS [4,7,34,60,61]. In this study, we focused on cross-reactivity of Roche Elecsys immunoassays for five steroid hormones (cortisol, DHEA sulfate, estradiol, progesterone, and testosterone).

Previous studies have demonstrated significant cross-reactivity of marketed cortisol immunoassays [13,61]. Our studies suggest that false positive cortisol measurements are most likely on the Roche assay during treatment with prednisolone or 6-methylprednisolone, in 21-hydroxylase deficiency due to elevated 21-deoxycortisol, or following metyrapone challenge due to 11-deoxycortisol. This is in accord with package insert data [19]. A study comparing cortisol measurement by immunoassay on the Siemens ADVIA Centaur XP analyzer versus LC/MS/MS demonstrated substantial positive bias of the immunoassay following metyrapone challenge, attributable to interference on the cortisol immunoassay from 11-deoxycortisol [61].

To our knowledge, other than package insert data [31], there is no published data on specificity of DHEA sulfate immunoassays. Our data suggests that cross-reactivity is likely not a major issue with the Roche Elecsys assay. In only two scenarios was cross-reactivity predicted to produce false positive DHEA sulfate values that would fall within the reference range: pregnenolone sulfate in pregnancy and 17-hydroxyprogesterone in 21-hydroxylase deficiency. In each of these cases, the contribution is likely minor even at maximally predicted interference levels.

A number of studies have compared immunoassay versus mass spectrometry for measurement of plasma/serum estradiol. Estradiol immunoassays often do not perform well relative to LC/MS/MS in measuring the lower end of estradiol concentrations found in males, a limitation likely related primarily to differing lower limits of quantitation between the methods [59,62,64,65]. There have been few reports of interferences with estradiol immunoassays. Negative interference by estriol has been reported in a study of the Abbott AxSYM estradiol immunoassay [66]. No significant interference by estrone or estriol was noted in a study of the Abbott Architect estradiol assay [64]. Our cross-reactivity studies suggest that clinically significant cross-reactivity with the Roche Elecsys Estradiol II is unlikely, similar to package insert data [37].

Compared to assays for estradiol and testosterone, there has been relatively little comparative study of progesterone immunoassays with mass spectrometry-based methods. A comparison of 12 progesterone immunoassays with gas chromatography/mass spectrometry (GC/MS) demonstrated high variability in specificity and sensitivity of the immunoassays compared to GC/MS [58]. In our study of the Roche Elecsys Progesterone II immunoassay, the most significant apparent progesterone concentrations were estimated to occur with 17-hydroxyprogesterone in patients with 21-hydroxylase deficiency and for 11-deoxycortisol following metyrapone challenge. Medroxyprogesterone and exemestane each may produce apparent progesterone of approximately 0.5 ng/mL. 5β-Dihydroprogesterone and allopregnanolone both may produce apparent progesterone concentrations in the range of 0.2 ng/mL. However, with the exception of 17-hydroxyprogesterone in patients with 21-hydroxylase deficiency, none of the other interferences likely produce high enough interference to cause diagnostic issues in women, where progesterone concentrations typically exceed 1 ng/mL. There is the possibility that these interferences could cause significant interference in progesterone measurements in males, although progesterone is typically infrequently measured in males. Doping with exemestane and other aromatase inhibitors has been reported in competitive athletes and others abusing anabolic steroids, primarily as a means to counteract gynecomastia and side effects related to aromatization of anabolic steroids by aromatase [57]. Our results raise the possibility that surreptitious use of aromatase inhibitors could interfere with some steroid hormone measurements by immunoassays.

Anabolic steroids were well-represented among compounds cross-reacting with the Roche Elecsys Testosterone II immunoassay with six of these compounds (boldenone, 19-norclostebol, dianabol, methyltestosterone, normethandrolone, and 11β-hydroxytestosterone) producing cross-reactivity of 5% or greater. Norethindrone, a progestogen commonly found in oral contraceptives, also produced strong cross-reactivity. Methyltestosterone, nandrolone, and norethindrone all appear capable of causing clinically significant false positives on the Roche testosterone assay, especially in females. However, interpretation of the clinical significance of the strong cross-reactivity of boldenone, 19-norclostebol, dianabol, normethandrolone, and 11β-hydroxytestosterone on the testosterone assay is hampered by lack of human pharmacokinetic data. We were unable to locate reliable serum/plasma concentrations for these compounds in humans. There is animal data for some of these compounds [51], mainly due to interest in detecting doping in animal sports such as horse racing, but it is difficult to know how well these data extrapolate to humans.

The results of this study raise interesting questions about the structural differences of diagnostic antibodies used for clinical measurement of steroid hormones. There have been a number of studies looking at the three-dimensional structure of antibodies that bind steroid hormones. A crystallographic study of two different estradiol antibodies revealed that antibodies with equally high specificity for estradiol relative to other steroids could, nonetheless, have markedly different amino acid sequence, ligand binding pockets, and ligand orientations [67]. Three studies of anti-testosterone antibodies demonstrated how directed mutagenesis could improve antibody specificity [68-70]. For steroid hormones, it would be of interest to compare and contrast the structure of antibodies used in different marketed immunoassays.

Our results using 2D-similarity to predict steroid hormone cross-reactivity show comparable findings to our previous studies predicting cross-reactivity of drug of abuse and therapeutic drug monitoring assays [14-17]. All compounds with strong cross-reactivity and most with weak cross-reactivity had 2D similarity values of 0.8 or higher to the target steroid molecule of the assay. Although there is some overlap in 2D similarity scores between compounds with strong or weak cross-reactivity and those with no cross-reactivity, use of a 2D similarity cutoff such as 0.8 would help identify compounds with high likelihood of showing strong cross-reactivity. Conversely, compounds with low 2D similarity (e.g., less than 0.6) are unlikely to show strong or even weak cross-reactivity. 2D Similarity calculations can thus be used to prioritize compounds for future immunoassay cross-reactivity studies for steroid hormones. This includes novel anabolic steroids used for doping or other as yet uncharacterized compounds, along with metabolites. There are some compounds with high similarity to the target molecule of the immunoassay which nonetheless have no cross-reactivity. An example is tetrahydrocortisone for the Roche Elecsys Cortisol immunoassay. It may be necessary to use three-dimensional methods such as pharmacophores or docking to understand why such compounds with strong similarity do not cross-react [17,53,71].

## Conclusions

Clinically significant cross-reactivity on steroid hormone immunoassays generally occurs with structurally similar drugs (e.g., prednisolone and cortisol immunoassays; methyltestosterone and testosterone immunoassays) or with endogenous compounds such as 21-deoxycortisol that can accumulate to very high concentrations in certain disease conditions. Compounds producing cross-reactivity in steroid hormone immunoassays generally have a high degree of structural similarity to the target hormone. Relative simple 2D similarity calculations can help triage compounds for future testing of immunoassay cross-reactivity.

## Abbreviations

DHEA: Dehydroepiandrosterone; ELISA: Enzyme-linked immunosorbent assay; GC/MS: Gas chromatography/mass spectrometry; HPLC: High-performance liquid chromatography; MS: Mass spectrometry; 2D: Two-dimensional.

## Competing interests

All authors (MDK, DD, CSM, JM, JLB, SE) declare that they have no competing interests.

## Authors’ contributions

MDK, JLB, and SE were involved in the study concept and design, analysis and interpretation of the data, drafting and revisions of the manuscript. DD, CSM, and JM performed the cross-reactivity studies. All authors have read and approved the final manuscript.

## Pre-publication history

The pre-publication history for this paper can be accessed here:

http://www.biomedcentral.com/1472-6890/14/33/prepub

## Supplementary Material

Additional file 1 **Cross-reactivity, similarity, vendor source, and assay details.** Spreadsheet with complete data on cross-reactivity, 2D-similarity calculations, vendor sources for compounds tested for cross-reactivity, and immunoassay details. Click here for file
